# Embracing Mechanobiology in Next Generation Organ-On-A-Chip Models of Bone Metastasis

**DOI:** 10.3389/fmedt.2021.722501

**Published:** 2021-09-01

**Authors:** Ellen E. Slay, Fiona C. Meldrum, Virginia Pensabene, Mahetab H. Amer

**Affiliations:** ^1^School of Molecular and Cellular Biology, Faculty of Biological Sciences, University of Leeds, Leeds, United Kingdom; ^2^School of Chemistry, University of Leeds, Leeds, United Kingdom; ^3^School of School of Electronic and Electrical Engineering, University of Leeds, Leeds, United Kingdom; ^4^School of Medicine, Leeds Institute of Medical Research, University of Leeds, Leeds, United Kingdom

**Keywords:** organ-on-a chip, breast cancer, metastasis, microenvironment, biomaterials

## Abstract

Bone metastasis in breast cancer is associated with high mortality. Biomechanical cues presented by the extracellular matrix play a vital role in driving cancer metastasis. The lack of *in vitro* models that recapitulate the mechanical aspects of the *in vivo* microenvironment hinders the development of novel targeted therapies. Organ-on-a-chip (OOAC) platforms have recently emerged as a new generation of *in vitro* models that can mimic cell-cell interactions, enable control over fluid flow and allow the introduction of mechanical cues. Biomaterials used within OOAC platforms can determine the physical microenvironment that cells reside in and affect their behavior, adhesion, and localization. Refining the design of OOAC platforms to recreate microenvironmental regulation of metastasis and probe cell-matrix interactions will advance our understanding of breast cancer metastasis and support the development of next-generation metastasis-on-a-chip platforms. In this mini-review, we discuss the role of mechanobiology on the behavior of breast cancer and bone-residing cells, summarize the current capabilities of OOAC platforms for modeling breast cancer metastasis to bone, and highlight design opportunities offered by the incorporation of mechanobiological cues in these platforms.

## Introduction

Metastasis is a complex and dynamic process. Breast cancer cells (BCCs) can remain dormant at the metastatic site, triggering relapse years after the treatment of the primary tumor. The presence of metastases in breast cancer patients decreases the 5-year overall survival rate to 27% (1). The development of physiologically relevant *in vitro* models that recapitulate key aspects of the *in vivo* mechanical microenvironment is urgently needed to advance our understanding of the biophysical forces that drive the various aspects of the metastatic cascade.

One reason why breast cancer metastases are challenging to model is the dynamic complexity of the tumor microenvironment (TME), which is increasingly recognized as a key factor in metastasis. During tumor progression, the extracellular matrix (ECM) undergoes substantial modification by cancer cells, surrounding cancer-associated fibroblasts and immune cells. These changes in the TME lead to the induction of angiogenesis and abnormal tissue function, contributing to metastasis (2). Advancing our understanding of the physical TME and developing physiologically relevant *in vitro* models of tumor complexity will increase the correlation between *in vitro* preclinical data and clinical outcome. Monolayer cell cultures are unable to simulate the complex TME and lack appropriate cell-cell and cell-matrix interactions (3). Whilst animal models allow for the consideration of cell-cell and cell-matrix interactions, they require extensive training, are ethically controversial, and do not always translate to human disease due to intrinsic genetic and physiological species differences (4).

**Figure 1:**
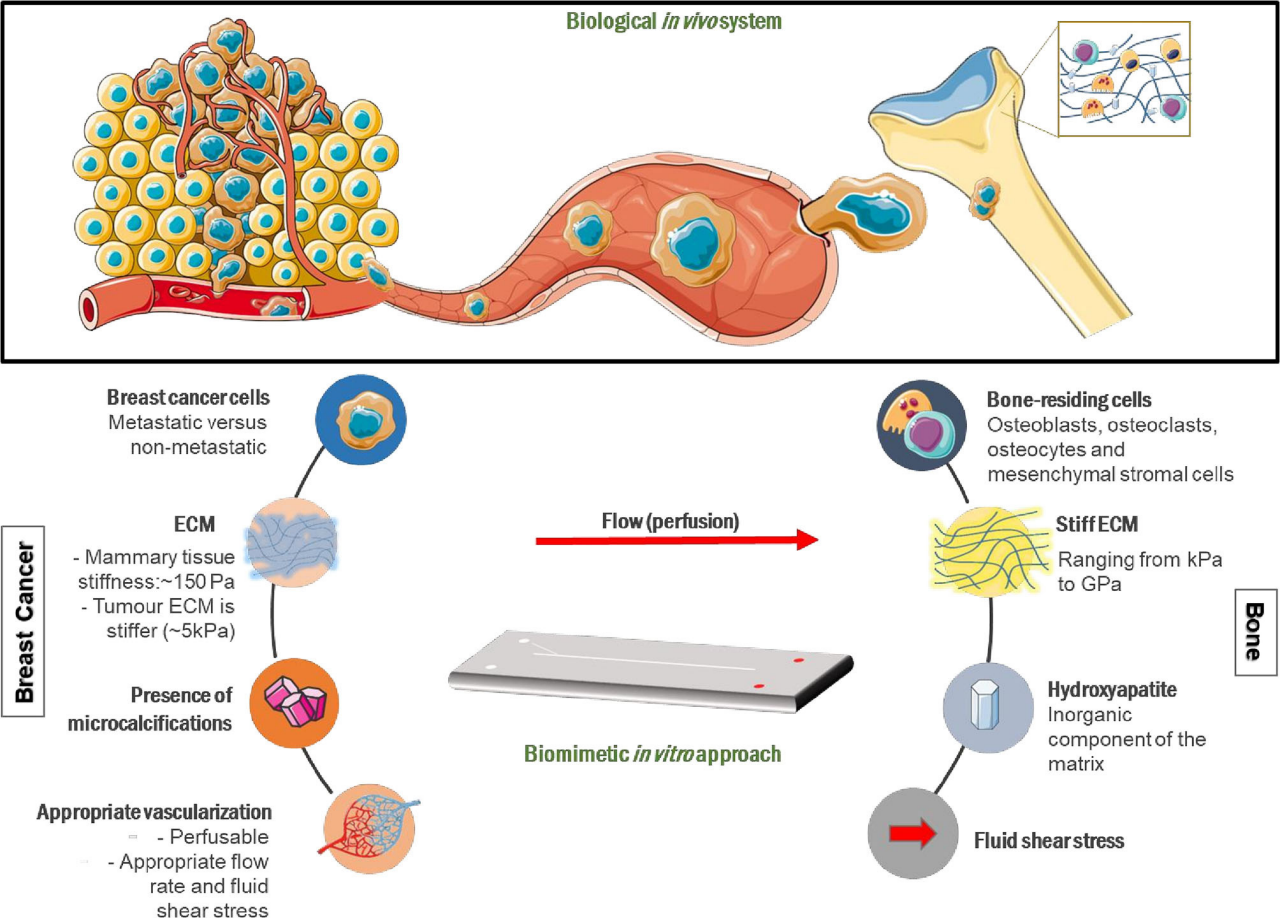
Mechanobiological design considerations for metastasis-on-a-chip systems mimicking breast cancer metastasis to bone. Breast cancer cells (BCCs) invade the extracellular matrix (ECM), intravasate into blood vessels where they circulate in the vascular system before extravasating into a secondary site, in this case bone, where they grow into secondary tumors *in vivo*. A breast cancer-mimicking compartment should be made up of appropriate breast cancer cells and a surrounding ECM that will generate physiologically relevant matrix stiffness. A bone-mimicking compartment will consist of bone-residing cells, including mesenchymal stromal cells, osteoblasts, and osteoclasts, as well as key bone ECM components, such as hydroxyapatite. The ECM components included will define the elasticity, topography and chemistry of the matrix. Interconnected, physiologically relevant fluid flow between compartments will allow cancer cell migration to the secondary site and the formation of suitable cytokine/growth factor gradients. Portions of the schematic were produced using Servier Medical Art (smart.servier.com).

Organ-on-a-chip (OOAC) platforms, also known as microphysiological systems, are three-dimensional (3D) *in vitro* models that contain cell-lined micro-channels, and are continuously perfused with culture medium (5). OOAC platforms are able to recreate the multicellular architecture and microenvironment of the tissue(s) being studied (6). However, the absence of immune, inflammatory and metabolic responses, limited culture span, and specific challenges associated with the representation of each organ with multiple connected tissue types (multi-OOACs) reduces their usability (7). In addition, the use of animal-derived matrices, such as the commonly used Matrigel, can lead to reproducibility issues (8). The potential of OOAC platforms to make an impact on academic research and industrial drug discovery is driving investment into new microfluidic approaches to model metastasis (9). Drug development necessitates well-defined, connected mixed-cell models that combine physiological flow and a supporting, physiologically relevant ECM. Functional OOAC platforms that allow communication between primary and secondary sites, each incorporating distinct, tissue-specific mechanobiological cues (Figure 1), will inform research and facilitate drug discovery by replacing the currently available poorly predictive models.

The strategic combination of tissue engineering strategies and OOAC platforms provides a path toward the construction of models that are truly physiologically relevant. While OOAC engineering is a growing field that is generating considerable scientific interest, especially in preclinical testing, the creation of environments that possess sufficient physiological complexity takes more than linking individual cell populations via microfluidics. This mini-review addresses the key question of how the design of OOAC platforms can be enhanced, summarizes current capabilities of OOAC platforms for modeling breast cancer metastasis to bone, and highlights the role of biomaterials in engineering physiologically relevant microenvironments and the design opportunities offered by the incorporation of mechanobiological cues.

## Role of the Physical Microenvironment As a Driver of Bone Metastasis

Tumor progression and metastasis is guided by cell interactions with the surrounding biochemical, cellular and mechanical cues. The ECM provides physical and biochemical cues for cellular proliferation and migration (10). Such interactions influence their behavior and consequently activates different signaling pathways (11, 12). Factors to consider when engineering physical microenvironments include matrix elasticity, topography, flow within the system and substrate chemistry (Figure 1).

### Matrix Elasticity

Breast tissue stiffness plays a role in the development of breast cancer (13), with mammary tissue undergoing up to 20-fold stiffening during tumor progression (14). Non-tumorigenic MCF-10A human breast epithelial cells cultured in a matrix of a stiffness of ~1 kPa has been reported to cause tumor-like development in these cells compared with those cultured on native mammary tissue stiffness of ~150 Pa, which did not develop a cancerous phenotype (15). Matrix stiffness affects genome accessibility, mediating the induction of malignancy via increased matrix stiffness (16). Stiffening of the ECM causes non-tumorigenic mammary epithelial cells to develop an invasive phenotype and activate mesenchymal gene expression (17). Matrix stiffening from <150 Pa to >3,000 Pa, at specific time points, was reported to promote epithelial-mesenchymal transition (EMT) in mammary epithelial cells (14). Matrix stiffness-induced EMT was reported in breast epithelial cells when grown on 5.7 kPa gels that mimic tumor stiffness, but not when cultured on gels that mimic the natural stiffness of breast tissue, via a TWIST1–G3BP2 mechanotransduction pathway (18). Malignant BCCs have been shown to adapt and survive on collagen matrices of different densities, whereas non-metastatic BCCs cannot (19). Cancer progression is also associated with significant softening of tumor epithelial cells relative to normal mammary epithelium, as detected by indentation-type atomic force microscopy (AFM), as well as broadening stiffness distribution of the TME (20). This demonstrates the dynamic nature of extracellular matrix remodeling in cancer progression.

The stiffness gradient within a tumor also affects the migration ability of BCCs. Anisotropic stiffness gradients caused by remodeling of the ECM triggers directional cell migration and increases BCCs ability to migrate and metastasize (21). Different types of BCCs respond differently to various levels of matrix stiffness. Triple-negative BCCs favor honeycomb geometries with greater stiffness, adenocarcinoma MCF-7 cells prefer mesh scaffolds with low elastic modulus, and pre-malignant cells favor aligned scaffolds with high stiffness and contact guidance (22). Additionally, it has been reported that rigidity sensing by healthy mammary cells is impaired in BCCs but remains active in normal mammary cells (23). This is further supported by work by Tse et al., who showed that compressive stress that accumulates during tumor growth can promote the migration of BCCs by stimulating the formation of leader cells and increasing cell–substrate adhesion (24).

Bone is a highly heterogeneous tissue. Elastic and viscoelastic measurements on cortical and trabecular bone have reported varying Young's/shear moduli in the GPa range (25–27). Differences within bone and bone marrow, determined by cell type and ECM components, also plays a significant role in bone metastasis *in vivo* (28). The endosteal surface is reported to be over 35 kPa, marrow sinusoids are more compliant (2–10 kPa), while the densely-populated central marrow is much softer (0.3 kPa) (29). Matrix elasticity has been shown to drive the differentiation of mesenchymal stromal cells (MSCs) (30). Furthermore, matrix stiffness modulates cellular stiffness during the differentiation process: The rate of increase in cellular Young's modulus during osteogenic differentiation has been positively correlated with matrix stiffness (31). Bone matrix elasticity also plays a role in the regulation of immune response by MSCs: Soft extracellular matrices maximize the ability of MSCs to produce paracrine factors that are implicated in monocyte production and chemotaxis upon inflammatory stimulation by tumor necrosis factor–α (TNFα) (32).

Matrix elasticity has also been shown to prime breast epithelial cells and regulate their collective migration (33). Epithelial cells primed on stiff matrices were reported to migrate faster, display higher actomyosin expression, form larger focal adhesions, and retain nuclear Yes-associated protein (YAP), even when cells had arrived at a softer secondary matrix. This showed that epithelial cells have memory of past matrix stiffness (33). Bone matrices with high elastic moduli, e.g., 380 kPa, can impart a bone-destructive phenotype in triple-negative MDA-MB-231 breast cancer cells via regulating integrin-β3 (*I*β*3*) and transforming growth factor-β (TGF-β) (34). Furthermore, parathyroid hormone-related protein (associated with tumor destruction of bone) and *I*β*3* expression increases with increasing substrate rigidity (34), promoting the progression and symptoms of bone metastasis. A reduction in matrix elasticity from 35 to 0.5 kPa was shown to increase MSCs homing to the breast TME (35). Migration of MSCs to the TME has been associated with an enhancement of tumor growth due to BCCs stimulating *de novo* secretion of the chemokine CCL5 from MSCs. CCL5 acts on BCCs to enhance their motility, invasion and metastatic capability (36). Migration rate of MDA-MB-231 cells toward a human trabecular bone explant was enhanced when cultured in stiffer gelatin hydrogels relative to softer ones, signifying an association between mechanical properties of the matrix and chemotactic signaling (37).

Human tissues exhibit viscoelasticity, which is a time-dependent response to loading or deformation. Viscoelastic materials display a combination of elastic and viscous characteristics (38). Breast cancer progression has been linked to variations in tissue viscoelasticity, with remarkable differences reported between benign and malignant tumors (39). Additionally, MSCs have been found to exhibit enhanced proliferation and osteogenic differentiation when cultured in 3D viscoelastic alginate hydrogels that exhibit faster stress relaxation. These effects were mediated by actomyosin contractility, adhesion ligand binding and ligand clustering (40).

### Topography

Topography is defined as the microscopic surface features that cells interact with, and is determined by the hierarchical structure of the ECM and roughness (41). Topography of the breast TME is a mixture of ridges, pores and collagen fibers, which makes it challenging to mimic *in vitro* (42). Topography regulates the expression and translation of human epidermal growth factor receptor type 2 (*HER-2)* (42), which is a predictive biomarker of the clinical efficiency of chemotherapeutic agents (43). While normal breast epithelial cells has been reported to show increased proliferation on topographically-patterned surfaces, defined by the presence of polystyrene beads of 23, 300, or 400 nm in diameter, estrogen receptor-positive MCF-7 breast cancer cell line showed lower proliferation (44). Furthermore, different BCC populations show distinctive behaviors on varying patterned culture substrates, dependent on cancer subtype. Metastatic MDA-MB-231 cells display uneven vinculin distribution when cultured on patterned substrates but not flat ones. On the contrary, non-metastatic MCF-7 cells do not exhibit this non-uniform vinculin distribution regardless of the substrates used. Significant difference in motility was also reported between the two cell lines (45).

Trabecular bone consists of a highly porous lattice network encompassing marrow and determines the topography of the majority of bone metastatic sites (29). Topography can be more dominant than biochemical cues and matrix elasticity in determining behavior of MSCs (30), and has been used to guide their differentiation (46, 47). Adhesion of MSCs was also shown to be bi-phasically regulated by interfacial roughness (48). In the context of bone remodeling, previous studies have reported the impact of surface topography on osteoclast differentiation and resorption (49, 50).

Guidance from matrix topography is a key regulator of metastasis. Nuclei of BCCs undergo extensive deformation when migrating through tight interstitial spaces (51). Topotaxis (directed cell migration guided by a gradient of topographical features) has also been suggested to influence the invasiveness of cancers (52). Benign and metastatic BCCs favor movement parallel to nano-ridges, showing greater speeds relative to flat surfaces. In contrast, asymmetric sawtooth structures create a unidirectional, cell type-dependent bias in the movement of BCCs (53).

### Flow

Shear stress, which is induced by fluid flow, contributes to proliferation and metastasis of tumor cells. Flow can either be through vasculature or through the ECM, the latter termed interstitial flow (54). Whilst different tissues support different flow rates, flow within solid tumors is generally much lower than in healthy tissue due to underdeveloped vasculature (55).

Flow within breast tissue has a significant impact on BCCs within the primary tumor. Higher flow rates promote EMT and increase cell metastatic capability (56), which promotes the conversion of BCCs into cancer stem-like cells and tumor-initiating cells by the suppression of extracellular signal-related kinase/glycogen synthase kinase 3β pathway (56). Interstitial fluid flow also generates mechanical forces that modulate tumor growth (57). Interstitial shear stress within breast tumor tissues is reported to create pressures of around 0.001 Pa (58) while circulating cancer cells can experience up to 3 Pa (59). When extravasation of cancer cell clusters (up to 20 cells) were investigated in a microfluidic device containing micro-channels with narrow constrictions of 5–10 μm, most were observed to migrate through the narrowest constrictions of 5 μm, unfolding into chain-like arrangements as they approached them and forming clusters as they exit. Clusters of over five cells displayed a migration velocity similar to the sum of resistances of the cluster's cells (60). Only 0.02% of circulating tumor cells survive to successfully undergo metastasis owing to anoikis, natural killer cells and mechanical damage by shear stress (61). Fluid flow also creates shear stress on endothelial cells, which leads to vasculature remodeling, cytoskeletal rearrangement and changes in the expression of transcriptional genes (62).

Interstitial fluid flow in tissues exposed to repeated mechanical loading, such as bone, induces variable levels of fluid shear stress of up to 3 Pa (63). Flow within bone causes osteocytes to release signaling factors, including nitric oxide and prostaglandin E_2_. These influence osteoblast behavior and trigger osteocytes to release receptor activator of nuclear factor kappa-B ligand (RANKL) and osteoprotegerin, the ratio of which is indicative of the amount of bone resorption (64). Laminar flow within bone is altered by compressive loading, muscle contractions, blood pressure changes and mechanical loading, and this generates varying shear stress on cells (65). Additionally, different cell types experience flow differently within the bone. Osteoclasts experience high flow and shear stress as they are directly exposed to flow through the lacunar-canalicular system (66). Osteoblasts, however, experience less flow and shear stress (67). Flow within the bone is also altered by the degree of mineralization, which changes the shear stress forces that cells experience (64).

Flow within bone has a significant impact on metastatic BCCs present, as increased bone remodeling can promote proliferation of BCCs (68). The “vicious cycle theory” states that osteoclastic bone resorption releases bone-derived growth factors, including TGF-β and insulin-like growth factor-1, which stimulates metastatic cancer cells to release osteoclast-stimulating factors, such as parathyroid hormone-related protein. These in turn causes further bone destruction through upregulated RANKL expression in MSCs and osteoblasts. This promotes the differentiation of RANK-expressing osteoclast precursor cells into mature osteoclasts, which resorb bone and so the cycle continues (69).

### Biomineralisation

Bone ECM comprises collagen, laminin, fibronectin, adhesive proteins, adipocytes, proteoglycans (70), and natural bone apatite (71). Around 60% of the dry weight of adult human cortical bone is mineral (71, 72). Mineral in bone is documented to be chemically similar to hydroxyapatite [HAp; Ca_10_(PO_4_)_6_(OH)_2_] although it tends to be less crystalline, more soluble, and highly substituted in the human body (73). HAp is known to interact with a variety of proteins, such as osteopontin, to support cell adhesion (74). Crystallinity, particle size, and substitution of HAp all affect cellular behavior. Highly crystalline HAp exhibits increased osteoconductivity (75). Smaller, less crystalline HAp enhances cell adhesion and proliferation whereas larger, more crystalline HAp particles enhance expression of the osteolytic factor interleukin-8 (IL-8) in BCCs (76), but not in the more benign MCF-7 cells (77). It has also been revealed that HAp has a role in the adhesion of BCCs (78) and causes BCCs to secrete higher levels of cytokines, including TGF-β and pro-osteoclastic IL-8, relative to non-mineralized scaffolds (79). IL-8 drives bone degradation (76) and results in the clinical symptoms of bone metastases, namely bone pain and pathological fractures (80).

It has been long understood that HAp, in the form of microcalcifications, is also found within breast tissue of breast cancer patients (81). The processes by which these microcalcifications form are largely unknown despite being positively correlated with BCCs mitogenesis (81). Microcalcifications with larger HAp particles have been more strongly associated with malignant breast cancer than microcalcifications made up of smaller particles (82). HAp has been implicated in upregulating the expression of several matrix metalloproteinases (MMPs), including MMP-2, MMP-9, and MMP-13, which promote BCCs migration due to decreased ECM elasticity (81). The existence of a direct relationship between mineral deposition and the ability of BCCs to metastasize to distant organs suggests a link between mineral deposition in the breast and metastasis. This has been suggested to be through the regulation of osteopontin expression (83).

HAp has also been implicated in promoting the colonization of metastatic BCCs in bone (84, 85). Although processes by which BCCs preferentially target bone and induce pathological remodeling remain unclear, there is increasing evidence that HAp is involved in this. HAp increases the expression of stromal-derived factor-1 (SDF-1) which promotes the activation of the migratory axis in BCCs thus directing their circulation to bone via increased signaling through the chemoattractant CXCR4 (85). Mineral-mediated changes in the collagen network were reported to result in increased cell motility (86). However, HAp inhibited directed migration of BCCs (86). These results suggest that mineralization of collagen fibrils reduces tumor cell adhesion, which may affect skeletal homing of disseminated tumor cells in the early stages of breast cancer metastasis (86).

## Mechanobiological Design Opportunities For Organ-On-a-Chip Platforms

OOAC platforms often consist of inlet and outlet ports for media to flow through and single or multiple chambers connected in different ways, allowing for interactions between various cell types (87). Microfluidics-based models have been designed in numerous ways to study the interactions of BCCs with the bone metastatic niche (Table 1). This approach has revealed that BCCs have a preference to extravasate to bone-specific microenvironments (93, 95), with flow-conditioned BCCs migrating further into the surrounding matrix relative to static controls (94). However, there are limitations to these microphysiological models. Many do not include vasculature, a distinct breast cancer compartment and/or a representation of the secondary site, and therefore cannot be effectively used to comprehensively study certain steps of the metastatic cascade (90, 92, 93). BCCs are commonly introduced directly to the secondary site rather than breaking away from the primary location, such that these models cannot be used to study extravasation (91, 94). Microfluidic approaches for investigating extravasation in metastasis have been reviewed previously (97). Another example is a microfluidics-based model consisting of vascular and tumor compartments only, which was used to investigate the formation of vascular vessels by endothelial cells (98). Metastatic tumor cells and tumor cell-conditioned media were shown to increase endothelial cell permeability and impair endothelial cell-cell junctions (98).

**Table 1:** Overview of microfluidic systems that study the role of the bone microenvironment in breast cancer metastasis, highlighting biomaterials used.

**Area of research**	**Relevant cells used**	**Cell growth surface**	**Key findings**	**Reference**
Role of bone cells and mineralization in adhesion of BCCs	Murine RAW264.7 (OCs), MDA-MB-231, MCF-7	3D HAp-mineralized, porous scaffolds made of PLG microspheres	HAp enhances BCCs proliferation and adhesion to the matrix. HAp upregulates the secretion of IL-8 by BCCs, which induces inflammatory response, angiogenesis and osteoclastic resorption.	(79)
Role of bone structure and mineralization parameters in adhesion of BCCs	hMSCs, MDA-MB-231, MCF-7	3D porous chitosan scaffolds containing HAp with different crystallinities, concentrations and grain sizes (micron/nano)	BCCs adhesion was increased in scaffolds containing 10% nano-crystalline HAp compared to those containing microcrystalline HAp. Coculture with hMSCs in HAp-containing scaffolds induced the upregulation of expression of the metadherin gene in BCCs (enhances metastatic potential and chemoresistance of BCCs).	(78)
	MDA-MB-231	Porous PVA scaffolds generated via foaming and freezing and then mineralized *via* immersion in modified HBSS for 14 days.	The greater the extent of mineralization of the scaffold, the greater the adsorption of serum proteins leading to higher BCC adhesion and proliferation.	(88)
Role of bone mineralization in adhesion of BCCs	MDA-MB-231	3D porous scaffolds containing HAp nanoparticles. HAp was aged for different lengths of time to increase crystalline development and added to the scaffold.	The smaller and less crystalline the HAp nanoparticles, the greater the adhesion of BCCs. Larger, more crystalline HAp particles stimulate more IL-8 production.	(76)
Role of bone structure in adhesion and survival of BCCs	hMSCs, MDA-MB-231	Scaffold was 3D printed with different geometries created: either large or small square or hexagonal pores. Printable ink consisted of HAp nanoparticles suspended in PEG/PEG-DA hydrogel.	Different geometries of 3D scaffolds influenced BCC adhesion, with the small square matrices displaying greater cell numbers than the others. BCCs were less responsive to 5-FU in 3D HAp scaffolds with their optimized geometry.	(89)
Role of bone cells in survival of BCCs	Human fetal osteoblast cell line (hOBs), MDA-MB-231	Porous constructs were 3D printed to allow for BCCs to form spheroids within the scaffold	Enhanced BCCs proliferation on HAp-containing matrices. BCCs co-cultured with hOBs directly affected the morphology, proliferation and IL-8 secretion by OBs.	(90)
Role of bone in colonization by BCCs	ECs, MSCs, MDA-MB-231	Decellularised bone matrix within a microfluidic chip	Interstitial flow promotes colonization of BCCs in the bone microenvironment and BCCs exposed to interstitial flow display a slow-proliferative state linked with chemoresistance.	(91)
	MDA-MB-231 and murine MC3T3-E1	Collagen-HAp composite in a PDMS device.	Osteoblastic tissue was invaded by BCCs, which eroded apical collagen and consumed the surrounding matrix.	(92)
Role of bone in extravasation of BCCs	hMSCs, HUVECs, MDA-MB-231	Cells grew in a PDL-coated PDMS channels, with a thin Matrigel layer coating the central media channel	BCCs extravasated significantly more in the bone-like microenvironment compared to collagen-only controls. Extravasation rate was associated with paracrine signaling via CXCL5 and CXCR2.	(93)
	hMSCs, OBs, HUVECs, MDA-MB-231	Cells mixed into a fibrin gel within a PDMS microfluidic device.	BCCs responded to the bone stromal cells via paracrine signaling, and this increased extravasation rate. Extravasation rate in bone-like environments was significantly higher relative to muscle-like microenvironments or controls.	(94)
	HDMECs, MDA-MB-231	Cells were seeded directly into a PDMS microfluidic device with no additional biomaterials	CXCL12 acts through CXCR4 on HDMECs to promote the adhesion of circulating BCCs, which promotes extravasation.	(95)
	hOBs, HDMECs, MSCs, HLF, MDA-MB-231	Multilayer microfabrication method used. Cells were seeded into rat tail collagen type I to introduce into PDMS microfluidic device	Bone-like microenvironment promoted extravasation of bone-tropic BCCs, suggesting OBs influence selective extravasation of BCCs.	(96)

*BCCs, breast cancer cells; FBs, fibroblasts; hMSCs, human mesenchymal stem cells; hOBs, human osteoblasts; ECs, endothelial cells; HDMECs, human dermal microvascular endothelial cells; HAp, hydroxyapatite; HLF, human lung fibroblasts; HBSS, Hanks' Balanced Salt Solution; HUVECs, human umbilical vein endothelial cells; IL-8, interleukin-8; OCs, osteoclasts; PLG, poly(lactic-co-glycolic acid); PEG, poly(ethylene glycol); PEG-DA, poly(ethylene glycol) diacrylate; PDL, Poly-D-lysine; PDMS, polydimethylsiloxane; PVA, polyvinyl acetate; 5-FU, 5-fluorouracil*.

There are a range of commercial microfluidic models that can be used to explore breast cancer metastasis to bone. These have been reviewed for their use in basic discovery research (9) as well as in drug testing and diagnostics (99). Commercialized devices are valuable as they can be tested across numerous laboratories and offer a standardized model. Examples include a microfluidic model used to investigate osteogenic differentiation *in vitro* using a flow rate ranging between 0 and 1,000 μL/h (100) and studying BCCs migration and proliferation in the presence of mechanical stimulation of osteocytes (101). However, other key substrate design parameters were not considered, such as topography. Another microfluidic model was used to recreate bone marrow cell populations with hydroxyapatite and a fluid shear stress range of 0.02–2 Pa, but did not recapitulate the heterogeneity of bone ECM stiffness and topography (102). A different microfluidics model was used to assess sensitivity to doxorubicin when cells are cultured in 2D vs. 3D (breast tumoroids) using physiologically relevant pressure and flow (550 μL min^−1^), highlighting the importance of a 3D environment (103). Lanz et al. proposed a high-throughput microfluidics-based platform with individual breast cancer spheroids for drug screening (104). This model utilized a gravity driven pump-free perfusion via a rocker system, generating fluidic shear stress of 0–0.3 Pa (9). Whilst this alone cannot be used to comprehensively investigate the metastatic process, it exemplifies the potential of incorporating both flow and varying ECM composition.

Whilst polydimethylsiloxane (PDMS) is the most common elastomer used for the development of OOAC platforms for research and fast prototyping, commercial models are often manufactured using hard plastics, such as polystyrene, to reduce production costs and time. The majority of these products have a solid structure, with the exception of lung-on-a-chip devices, where the device structure includes an elastic silicone membrane that can be stretched to create periodic mechanical forces and adapts to fluid stress, thus mimicking tissue dynamics (5). However, polymers used for the fabrication of membranes typically used in OOAC platforms are flat and do not account for tissue surface morphology. The degradability of these elastic polymers is another important consideration, as this determines possible changes in membrane properties over time and potential cytotoxicity issues that may arise.

To expand the potential of these devices, more sophisticated engineered environments must be created that embrace biomimetic topographical, elasticity and chemical engineering design aspects. Figure 1 presents various mechanobiological parameters that could be incorporated into OOAC platforms to allow the investigation of the complex metastatic pathway. There have been recent advances in OOAC platforms with multiple linked organs (105–108). Many challenges remain, including choice of culture medium across organ representations, ensuring optimal organ-specific time points in protocols, and creating approaches for on-chip imaging and non-invasive sample analysis over extended time periods.

### Engineering Matrix Elasticity

Studies into the influence of matrix elasticity on breast and bone tissues gives rise to fundamental knowledge that can be applied to OOAC platform design. Methacrylated hyaluronic acid hydrogels have been used to mimic the stiffening of breast tissue in microwell plates to model epithelial-mesenchymal plasticity and the metastatic capability of BCCs (14). Polyethylene glycol (PEG) have also been introduced into collagen gels to alter their stiffnesses without altering their microstructures (109). These can be applied within OOAC models, where PEG is mixed with cells and pre-gelled collagen solution before inserting into the platform. Gel formation would then be induced by changing pH. PEG changes the non-covalent forces that are responsible for collagen self-assembly, so varying the concentration of PEG can vary the stiffness of collagen gel (109). With UV-crosslinked hydrogels, such as PEG and its derivatives, stiffness can be tailored by modifying macromer content and molecular weight (30). Another example are PEG-based hydrogels containing enzymatically degradable peptide sequences with varying concentrations of the integrin ligating peptide RGDS, and the non-degradable, co-monomer N-vinyl pyrrolidinone (NVP) (110). This allows tuning of cross-linking density and degradation, and has been shown to generate different phenotypic states in BCCs (110). However, changes in the content of the monomer and crosslinking will alter the porosity of the matrix; this impacts protein tethering, which in turn influences stem cell differentiation (111). Whilst this is an effective method for the inclusion of customizable matrix stiffness within OOAC platforms, it does not permit dynamic changes in stiffness of the TME as seen in cancer progression.

Conventional hydrogels offer inadequate dynamic signals, such as spatial or temporal control of formation/degradation, and the mechanical strength required to mimic bone stiffness. One way to mimic the dynamic TME is to use tunable hydrogels, which allow flexible control of the biophysical features of the gels in question to study how changes in matrix stiffness affect a cell population over time. One example is photodegradable hydrogels. Light-responsive biomaterials are of interest as an approach for spatiotemporal regulation of cell dynamics. These can be modified in a controlled manner by illuminating with light of specific intensity and wavelength (112). Progress has been made in the synthesis of dynamically tunable photo-responsive biomaterials (113, 114). For 4D cell culture (where 3D culture substrates are used to present changing biophysical or biochemical properties over time), chemically-decorated hydrogels have been fabricated with a combination of photochemistry and other orthogonal click reactions (115, 116). Temporal and spatial control of *in vivo* presentation of cell-adhesive RGD peptides has also been reported using a protecting group that can be removed via transdermal light exposure to activate the peptide (117). Temporal stiffening of substrates is key to mimicking breast cancer progression *in vitro*, and was reported to induce invasion from MCF-10A mammary acini (15). Innovative engineering of the mechanics and multi-scale architecture of tunable hydrogels is needed to achieve physiologically relevant features for *in vitro* modeling.

Cells often exhibit directed migration in response to the rigidity of the surrounding environment, migrating toward stiffer regions. This mechanosensitive behavior is termed durotaxis. Matrix elasticity gradients can be created to mimic *in vivo* stiffness gradients by exploiting chemical and mechanical gradients within OOAC platforms. This has been previously used to investigate haptotactic behavior in neurite growth, where a matrix elasticity gradient was created across an H-shaped collagen gel to investigate neurite growth (118). Additionally, matrix stiffness of the secondary site needs to be considered as bone ECM is much stiffer than breast tumor ECM.

### Incorporation of Topographical Features

A number of attempts have been made to incorporate topography in microfluidic devices. Replication of bioinspired surfaces with tunable 3D topography has been achieved using microinjection compression molding with novel dual-layer molds to create open microfluidic devices (119). Yang et al. fabricated nano-patterned surfaces using photolithography and electron beam lithography and stitched them together to assemble PDMS-based microfluidic platforms (120). Integration of patterned electrospun fibers into microfluidic systems to create aligned and random patterns in PDMS microfluidic chips was also used to create a complex microenvironment to mimic that *in vivo*, as validated by neural stem cell alignment (121). Another method to integrate topography is by surface patterning, for example PDMS grates. Grates with widths from 2 to 4 μm have been shown to cause MDA-MB-231 cells to align along the grating length and to increase the extension and spreading of these cells compared to planar controls (122). Topography can also be exploited in OOAC platforms to mimic BCCs migration seen *in vivo*. For example, nanoscale ridges have been shown to enhance the movement of benign and metastatic BCCs relative to flat surfaces (53).

Polyacrylamide hydrogels with different patterns, including spirals, stars, and squares, have been employed to study the concept of ‘topographical memory', showing that topography can affect histone modifications and prime cancer cells to a tumorigenic state (123). Different shaped fibers have been tested for their topographical effect on BCCs, with curved polystyrene fibers causing MDA-MB-231 cells to exhibit sensitivity to curvature, in terms of eccentricity (measurement of protrusion width), compared to flat ribbons (124). Varying the diameter of type I collagen fibers has also been investigated, where BCCs exhibited ~20% increase in cell spreading on type I collagen fibers with a diameter of 850 nm compared to 550 nm. Cell invasiveness also increased with fiber diameter, although proliferation was unchanged (125). This shows that cell response and tumor invasiveness are dependent on substrate surface patterns and collagen fiber diameter, and highlights the importance of considering topography of the microenvironment created within OOAC platforms.

Constructing appropriate topographical cues within metastasis models is essential, in terms of both the surfaces which cells have to travel over and the environment which they must travel through. The dimensionality, length and scale of the physical microenvironment has been reported to influence MDA-MB-231 triple-negative carcinoma cell signaling and decision-making at intersections of micro-contact printed lines (126). Cells confined in narrow micro-channels, and therefore had fully explored their microenvironment, favored entry into wider branches at bifurcation points, whereas cells in wider channels made pore size–independent decisions (126). Cancer cells traveling through ECM pores, modeled using a microfluidic device containing micro-channels of varying widths (3–50 μm), was shown to prevent the normal increase in cellular size during cell cycle progression, resulting in a reduction in the frequency of cell division and an increase in the frequency of abnormal division events (127). Pore size reduction within collagen-PEG mesh networks causes BCCs to exhibit reduced cell spreading, increased cell-cell adhesion protein expression, larger cell aggregates and triggers morphogenesis (128).

One way of incorporating topographical cues within OOAC platforms to mimic topographical variations sensed by metastatic BCCs would be to use temperature- or pH-sensitive hydrogels. Tunable topographies can be created using shape-changing, stimuli-responsive polymers or self-folding films to provide cells with an appropriate spatial environment for cells (129). Thermoresponsive hydrogels have been created with poly(*N*-isopropylacryalmide)-based copolymers (130–132) and exhibit low critical solution temperature behavior, swelling in water at lower temperatures and contract with increasing temperature (129). Temperature-sensitive hydrogels can be used for the fabrication of bilayers, which allow reversible folding and unfolding at low and elevated temperature, respectively, and have been used for cell encapsulation (130, 131). The topography of these hydrogels can be altered: Swelling will stretch the culture surface by expanding it. By utilizing shape transformation of these tunable hydrogels there is greater potential for the creation of dynamic structures with high resolution, which are difficult to achieve by other biofabrication techniques, such as bioprinting (129). Topography can also be created and controlled by utilizing culture substrates such as polymeric microparticles within OOAC platforms. Topographically-designed microparticles have been used to drive MSCs down an osteogenic lineage without the use of exogenous osteo-inductive factors (47). In addition, surface roughness is important in bone OOAC platforms as osteogenic differentiation can be enhanced in MSCs by culturing them on growth surfaces with intermediate roughness (48). This is controlled through roughness-regulated expression of YAP (48). Substrate roughness is another design parameter to consider. Sandblasting of the surface of poly(methyl methacrylate) films with alumina grains has been used to alter roughness, resulting in increased adhesion and migration of vascular cells with higher surface roughness. Increased cell adhesion was attributed to higher adsorption of proteins, such as fibronectin and collagen I, on the surface of the films (133).

Topography can also be controlled on the surface of the membranes used within OOAC platforms. These membranes are often suboptimal, as they are typically flat and do not replicate the shape or surface morphology of a tissue (134). Microfabrication techniques can be applied to create porous membranes with suitable porosity, shape and surface morphology to match the requirements of the tissue being modeled. Porous membranes with micron-sized features can be made by electrospinning (135). Soft lithography and thermoforming are used to prepare porous, micro-structured membranes for other applications, where thermoforming is combined with ion track etching to create microstructures and pores, respectively (134). Phase separation micro-molding is able to create patterns and pores in membranes in one step by inducing phase separation (136).

### Flow Rate

Flow of cell culture media within OOAC platforms generates pressures and oxygen gradients which affect cell behavior and survival (65). As there are different flow rates within the body, different organs modeled within OOAC platforms require different flow rates. There are several ways to recreate flow in these devices, including the use of peristaltic pumps, syringe pumps, or rocker plates. These different methods can create significantly different flow rates, with peristaltic pumps creating the strongest rates and rocker plates the slowest ones (137). The technique for perfusing an OOAC platform therefore needs to be carefully selected for each organ component.

Microfluidic channels filled with gels can be used to study the effects of interstitial flow. Haessler et al. used this technique to study the effects of 10 μm s^−1^ interstitial flow on MDA-MB-231 cells (138). Gel-free systems or channels coated in a thin layer of gel only can be used to study laminar flow. For example, a channel can be lined with gelatin before allowing fluid to flow through the channel with endothelial cells to form a single channel blood vessel (139). Lanz et al. seeded metastatic triple negative BCCs in an artificial ECM under static and dynamic conditions, with cells displaying a proliferation rate of 80% in the perfused system compared to 60% in the static one (104). Pradhan et al. fabricated a high and low perfusion chip to mimic cancer-ECM-endothelial interactions. This showed that BCCs elongated forming colonies similar to those formed *in vivo* in high perfusion regions, whereas BCCs appeared rounded, relatively dark and unhealthy in low perfusion region, possibly indicative of a quiescent state due to lack of nutrient availability (140). Extreme flow rates are undesirable within breast OOAC platforms, as only peripheral and circulating BCC should be exposed to high flow rates (56). BCCs cultured within 3D ECM are more representative of *in vivo* BCCs, displaying greater proliferation, invasiveness, chemoresistance and higher plasminogen activator urokinase signaling when subjected to interstitial flow than cells in static conditions (141).

Flow within bone and vasculature representations is also of great importance. Fluid shear stress governs the binding of tumor cells to endothelial cells. Breast cancer cells were reported to not adhere to endothelial cells directly under low shear stress of 0.5–2 dyn cm^−2^, instead forming a tumor-monocyte complex before binding to endothelial cells (142). MSCs grown on a bone-on-a-chip system under flow (30 μL/h; 0.346 mPa) showed improved survival and proliferation relative to cells grown under static conditions (100). Fluid shear stress was reported to enhance osteogenic differentiation in an osteogenesis-on-a-chip device, but was not sufficient to induce it on its own (143). Conditioned medium from oscillatory flow-stimulated osteocytes significantly increased migration and reduced apoptosis of BCCs (144). In another study, MDA-MB-231 BCCs were cultured inside a microfluidic channel lined with human endothelial cells and adjacent to another channel containing osteocyte-like cells. Physiologically relevant oscillatory fluid flow (1 Pa, 1 Hz) was applied and hydrogel-filled side channels allowed real-time extravasation to be observed. The applied fluid flow induced intracellular calcium responses in osteocytes (3.71-fold increase), with extravasation distance significantly reduced by mechanically-stimulated osteocytes relative to static osteocytes (145). Additionally, a physiologically relevant perfusable vasculature to investigate intravasation, migration, and extravasation, cannot be achieved without flow (62) and shear stress is required to maintain the system (146). Once vasculature has developed, flow continues to have a vital role in decreasing the permeability and increasing the stability of vessel walls within the vasculature (146).

### Chemistry and Biomineralisation

Materials used to create OOAC platforms, such as elastomers, are commonly formed from acrylate monomers, which can adsorb different amounts of fibronectin. This affects cell attachment (147) and integrin signaling, which can promote osteoblast differentiation (148). Furthermore, elastomers can affect the composition of culture medium. PDMS, for example, has been shown both to adsorb small molecules and release other chemicals into the device (149). This can be overcome by priming devices with ECM components such as collagen, or by using a lipophilic coating (150).

A key part of the design of the bone component of a metastasis-on-a-chip platform is the inclusion of a mineral component. HAp-coated ceramic scaffolds with MSCs and hematopoietic stem/progenitor cells have already been successfully integrated into a microfluidic device (102). HAp has been shown to affect viability, proliferation and cytoplasmic volume of metastatic colorectal and gastric cancer cells when incorporated in a bone-mimicking OOAC model (151). HAp can be precipitated in bulk solution as anisotropic needles, and 50 nm pores support the formation of HAp rods that are comparably oriented to native mineralized collagen fibrils (152). If precipitated within the confines of cylindrical pores that offer physiologically relevant gap regions (>100 nm diameter), polycrystalline HAp rods are formed (152, 153).

The inclusion of HAp must be carefully considered, as it may have a negative impact on vascularization. While Jusoh et al. reported that HAp has a positive effect on sprouting angiogenesis (154), increasing HAp concentration was reported to result in a decrease of the number of blood vessel sprouts, with angiogenesis sprouting length displaying a biphasic response to increasing HAp concentration (151).

It has been demonstrated that epidermal growth factor and CXCL12 gradients cooperatively increase tumor cell motility and are important in regulating cell migration in 3D environments (155). Chemical gradients can be included within OOAC platforms by introducing these molecules through microfluidic gradient generators (156). Shared media and interconnecting chambers allow media exchange within the device and permit natural gradients to form. In this way, cytokines and growth factors released from cells in secondary locations, such as MSCs in bone microenvironment, can spread through the OOAC to create physiologically relevant gradients.

Components of the ECM can be studied within OOAC platforms. One example of this is a compartmentalized platform developed by Sung et al., which enabled the analysis of the intrinsic second harmonic generation signal of collagen. This offered a label-free assessment of collagen remodeling in the model (157).

## Concluding Remarks and Future Directions

Breast cancer metastasizes to bone, lung and brain tissues, whereas prostate cancer, for example, mainly metastasizes to bone. This suggests that cancer cells respond to signals from the secondary site, leading to preferential migration. An ideal model of metastasis will allow the full metastatic cascade to be modeled, permitting the observation of cell dissemination from a primary tumor to secondary sites. Such a model is critical for understanding cross-organ communication that may lead to metastasis and for identifying biomarkers of cancer metastasis, including circulating tumor DNA/cells, which play a key role in metastasis. The evident obstacle to the investigation of metastasis using OOAC platforms is the successful recreation of the broad, dynamic range of the physiological complexity of the microenvironments at the primary and secondary sites, and the complex paracrine signaling implicated in metastasis. Multi-OOACs platforms have been developed to allow the study of systemic processes, which has been recently reviewed (158, 159). Numerous coupling arrangements have been proposed for building such “body-on-a-chip” approaches, which present challenges such as the lack of appropriate vasculature modeling, which is often simply represented by tubing, and the difficulties associated with optimizing the circulating media composition to ensure the long-term viability of multiple different compartments.

Physical microenvironments can be determined by the biomaterials used in the manufacturing of the device, such as PDMS, as well as the choice of biomaterials integrated within the microfluidic culture system. Combinatorial screening platforms have been recently developed that can help identify the most appropriate micro topographically-patterned polymers (160). OOAC platforms that incorporate dynamic mechanical stimuli are ideal for creating microenvironments to replicate *in vivo* cell behavior. Existing models do not accurately mimic the *in vivo* biomechanical environment, but instead focus on individual stimuli, most commonly flow rate. The inclusion of tunable hydrogels and other polymeric scaffold systems together with physiologically relevant flow rates are critical to achieve tissue-specific, dynamic cellular systems that capture the key aspects of the metastatic cascade. By combining current chip platforms with increased awareness and consideration of mechanobiology, OOAC platforms can be used to advance knowledge and understanding of metastasis and ultimately lead to the development of a more effective drug discovery pipeline for bone metastatic cancers.

## Author Contributions

ES and MA reviewed and evaluated the literature and drafted the manuscript. VP and FM critically revised the manuscript. All authors approved the submitted version.

## Conflict of Interest

The authors declare that the research was conducted in the absence of any commercial or financial relationships that could be construed as a potential conflict of interest.

## Publisher's Note

All claims expressed in this article are solely those of the authors and do not necessarily represent those of their affiliated organizations, or those of the publisher, the editors and the reviewers. Any product that may be evaluated in this article, or claim that may be made by its manufacturer, is not guaranteed or endorsed by the publisher.
